# Sign-tracking bias is associated with the inhibition of motor response to appetitive food cues

**DOI:** 10.1162/IMAG.a.1118

**Published:** 2026-02-03

**Authors:** Hugo Najberg, Malika Tapparel, Joel N. Holmann, Lucas Spierer

**Affiliations:** Laboratory for Neurorehabilitation Science, Medicine Section, Faculty of Science and Medicine, University of Fribourg, Fribourg, Switzerland

**Keywords:** Pavlovian learning, sign-tracking, Go/NoGo, response training, ERP, electrical neuroimaging, food

## Abstract

Response training, such as with the Go/NoGo task, reduces the value of trained items by requiring participants to repeatedly inhibit their responses to appetitive cues that typically elicit approach tendencies. This devaluation is thought to reflect a reduction in motivational conflict between the cue-driven approach and task demand for response withholding. In this registered report, we examined whether individual differences in Pavlovian learning style, measured via a sign-tracking bias during a separate Pavlovian conditioning task, are associated with the topography of event-related potentials recorded during a food-related Go/NoGo task. We also assessed whether this bias correlates with participants’ reaction times and commission errors during the task. We found a covariance between sign-tracking bias and pre-training topographic electrophysiological responses during the P3 ERP component, but no association with training-induced plastic modification. Additionally, we found positive evidence for an absence of relationship between sign-tracking bias and behavioral responses at pre-training (*r* = 0.0, BF_01_ > 3), and weak evidence for its absence post-training (*r* = 0.1, BF_01_ < 3). These findings indicate that sign-tracking bias modulates the activity of a specific brain network involved in inhibition of responses to appetitive cues. However, the absence of modulation induced by training suggests that food Go/NoGo training operates through pathways independent of the sign-tracking bias.

## 1 Introduction

Food cue valuation develops partly via Pavlovian learning processes, through their association with affective responses. These processes, in combination with other mechanisms such as instrumental learning and contextual factors, contribute to the formation of eating habits (Cartoni et al., 2016; Lovibond & Colagiuri, 2013; Martin-Soelch et al., 2007; Morris et al., 2015; Watson et al., 2014). Recent lines of research have demonstrated that food cues can also be “devalued” when associated with aversive outcomes, such as response withholding during food Go/NoGo (GNG) training (Veling et al., 2017). The aim of the present study is to investigate whether differences in Pavlovian learning tendencies, measured by a Pavlovian conditioning task, are associated with the effect of food GNG training, both at the electrophysiological and behavioral levels.

Food GNG training is a promising way to restore healthy eating habits, both in healthy and clinical populations (e.g., obesity, diabetes; Stice et al., 2016), by inducing food cue devaluation through the repeated inhibition of appetitive food stimuli (Allom et al., 2016; Jones et al., 2016). The repeated association of the food cues with response inhibition results in reductions of the trained item’s liking (Chen et al., 2016; Lawrence et al., 2015; Najberg, Rigamonti, et al., 2021), in turn reducing their consumption (Adams et al., 2017; Lawrence et al., 2015; Najberg et al., 2023) with the diminution of explicit liking being the most robust effect found in the literature (see for reviews: Allom et al., 2016; Carbine & Larson, 2019; Jones et al., 2016). The devaluation is thought to result from an updating of the value of the cues eliciting a motivational approach response when paired with response inhibition during training (Veling et al., 2022), not because of a top-down effect of inhibitory control training (Veling et al., 2017) but because response inhibition is interpreted by the participants as avoidance (Chen & Van Dessel, 2024). Accordingly, the devaluation constitutes a way to reduce the conflict between the appetitive nature of the cue (prompting an approach) and the inhibition response required by the training task.

Given that food GNG training may operate through Pavlovian learning processes, as appetitive cues are devalued following repeated pairings with “negative” motoric responses, individual differences in how people respond to such learning processes should influence the response to food GNG training.

Indeed, there is significant interindividual variability in the way individuals react to Pavlovian conditioning: ‘sign-trackers’ (STs) tend to focus on and attribute motivational value to reward-predictive cues, whereas ‘goal-trackers’ (GTs) focus on the relationship between the cue and its associated outcome (Flagel et al., 2011; Wuensch et al., 2021). This so-called sign-tracking bias can be assessed using eye-tracking during a Pavlovian conditioning task, where neutral stimuli are repeatedly paired with monetary rewards (Garofalo & di Pellegrino, 2015). This task results in the participants being able to explicitly associate the neutral stimuli (i.e., fractals) to their affective rewards, assumed to result from Pavlovian conditioning. Research indicates that STs exhibit longer fixations on cues predicting positive rewards than GTs. At the neurophysiological level, STs show greater activity in subcortical areas associated with affective and reward responses such as the ventromedial prefrontal cortex, the amygdala and the nucleus accumbens (Schad et al., 2020).

Building on evidence that sign-tracking reflects sensitivity to Pavlovian cue–reward associations, we tested whether individual differences in sign-tracking bias predicted how participants process appetitive food cues during and after GNG training.

We investigated whether sign-tracking bias predicts variability in training-related electrophysiological and behavioral responses (H1, H2). We expected the following: H1a) participants’ sign-tracking bias is associated with baseline neural responses to correctly inhibited appetitive food cue. H1b) Sign-tracking bias is associated with changes in brain activity induced by a 2-week food GNG training regimen. For both H1a and H1b, confronting the latency of the modulation with current temporal models of stimuli processing during an GNG task enabled us to determine the underlying mechanisms of action (see Table 1). H2a) Participants’ sign-tracking bias is associated with baseline behavioral responses during a food GNG task, with STs exhibiting shorter reaction times and higher commission error rates to appetitive food cues than GTs. H2b) After 2 weeks of food GNG training, participants’ sign-tracking bias is associated with the change in behavioral responses during a food GNG task. Details on the hypotheses and the expected mechanisms of action at each latencies/index are reported in Table 2.

**Table 1.** ERP latencies and associated non-exclusive underlying mechanisms in H1.

Estimated Latency Range (ms)	ERP Component	Functional role indexed by the ERP component	Significance under H1a (STs > GTs)	Significance under H1b (no direction for STs vs. GTs) *
<100	P1	Emotional and attentional salience for appetitive food cues (bottom-up attention orienting) (Antal et al., 2000; Nijs et al., 2009).	STs would have a stronger emotional and attentional salience toward appetitive cue during the food Go/NoGo task.	The training would induce a larger change than before training in emotional and attentional salience toward the trained appetitive NoGo cues.
100–200	N2	Solving of the inhibition response conflict in front of appetitive cues (De Pretto et al., 2019; Manuel et al., 2010).	STs would show a stronger conflict between the need for inhibition and the appetitive stimuli during the food Go/NoGo task.	The training would induce a larger change than before training in the inhibition conflict to solve when presented to the trained appetitive NoGo cues.
200–350	P3	Implementation of the response inhibition command toward appetitive cue (Antal et al., 2000; Nijs et al., 2009).	STs would need to engage a stronger inhibition to appetitive cue during the food Go/NoGo task.	The response inhibition would change more after than before training in front of the trained NoGo cues.

* For H1b, the direction of the hypothesized modulations induced by training was not predicted, as a stronger change can manifest both as a negative (i.e., less activity to obtain the same results) or positive difference in signal (i.e., more activity to reach better results; Spierer et al., 2013).

**Table 2.** Study design.

Question	Hypothesis	Sampling plan	Analysis plan	Interpretation given to different outcomes	Observed outcomes
There is an association between the functional mechanisms underlying food GNG training and Pavlovian learning.	H1a) Participants’ sign-tracking bias is associated with baseline neural responses after the correct inhibition of an appetitive food cue. These differences can be expressed at baseline in: • the P1 ERP component (< 100 ms post stimuli onset) for emotional and attentional salience for appetitive cues. • The N2 ERP component (100-200 ms) for conflict resolution demands. • The P3 ERP component (200-350 ms) for difficulty inhibiting responses to appetitive cues.	47 participants based on behavioral rationales (see Sampling plan section).	Time-wise TANCOVAs on the pre-training ERPs using the sign-tracking bias as the covariate. The ERP is time-locked around the onset of trials presenting correct inhibition during the GNG task. The Global Field Power (GFP) of the average ERP across participants was used as the TANCOVAs’ statistic.	If at least 12 consecutive time-frames (TFs; i.e., ca. 12 ms for a sampling rate of 1,024 Hz) have *p*-values below 0.05, the ERP modulations would be considered significant. If we fail to reject the null hypothesis for the relevant ERP, we could conclude that there are no baseline differences in neural processing (H1a) or no significant training-induced neuromodulations (H1b) associated with sign-tracking bias for the respective posited mechanism of interest during food GNG training.	Confirmed: Sustained significant covariance between sign-tracking bias and pre-training ERPs to successful inhibition of appetitive cues during the P3 component.
	H1b) The sign-tracking bias is associated with the functional plasticity induced by a 2-week food GNG training regimen. These differences should be expressed during the same ERP components as above. No directions in these modulations are expected.		Time-wise TANCOVAs on the within-subject factor Session (pre- vs. post-training) ERPs using the sign-tracking bias as the covariate (the sign-tracking bias is only assessed once for each participant). The ERP is epoched around correct inhibition trials at the GNG task. The GFP of the average ERP across participants were used as the TANCOVAs’ statistic.		Disconfirmed: No covariance of sign-tracking bias to ERP modulations induced by training, despite significant training-induced ERP changes during the N2 and P3 components.
There is a behavioral link between the mechanisms underlying food GNG training and Pavlovian learning.	H2a) Participants’ sign-tracking bias is linked with baseline behavioral responses during a food GNG task, with STs exhibiting shorter reaction times and higher commission error rates to appetitive food cues than GTs. • Shorter reaction times would express stronger emotional and attentional salience for appetitive cues. • Higher commission error rates would express higher demands for response inhibition toward appetitive cues.	47 participants based on a priori power analysis with an *r* of 0.4, alpha of 0.05 and power of 0.90	For both H2a and H2b, Pearson correlations between the sign-tracking bias and 1) the participants’ average reaction time of correct responses, and 2) the participants’ commission error rate (number of motoric errors divided by the total number of “NoGo” trials). Analyses are performed at baseline for H2a (i.e., outcomes measured during the pre-training in-lab Go/NoGo task), and on pre- post-training deltas training for H2b (i.e., outcomes at the post-training in-lab Go/NoGo task subtracted to their respective pre-training value for each participant). The sign-tracking bias is only assessed once for each participant. Bayes Factors against the null model was computed to interpret the null hypothesis.	If *p* < 0.05 on the correlation between the sign-tracking bias and the average response time of correct responses (at either baseline or pre- post- training), we could conclude that STs have a stronger emotional and attentional salience for appetitive cues. If *p* < 0.05 on the correlation between the sign-tracking bias and the commission error rate (at either baseline or pre- post-training), we could conclude that STs have higher inhibition conflict to solve. If BF_01_ > 3, we can conclude that the sign-tracking bias is not associated with behavioral responses in food GNG training for the respective posited mechanisms.	Disconfirmed: No relationship between sign-tracking bias and behavioral GNG performance pre-training.
	H2b) After 2 weeks of food GNG training, participants’ sign-tracking bias is linked with the change in behavioral responses during a food GNG task. These differences should be expressed with the same index as above.				Disconfirmed: No relationship between sign-tracking bias and pre- post-training deltas of the behavioral GNG performance.

The electrophysiological hypotheses were assessed using electrical neuroimaging analyses of the ERP (Murray et al., 2008) collected during a GNG task recorded before and after a 2-week response training intervention on the food cues (Carbine et al., 2018). We used a Topographic Analysis of Covariance (TANCOVA), a statistical approach used to analyze EEG data by identifying how scalp field potentials covary with external variables over time (Koenig et al., 2008). This method leverages the additive nature of electric fields produced by brain sources, ensuring the covariance of scalp potentials across sensors reflects the underlying intracerebral sources associated with an external variable (i.e., the participants’ sign-tracking bias). By using global field power as the test’s statistic and employing resampling statistics (see method section), TANCOVA detects the statistical significance of these covariations. It has been successfully applied to study relationships between brain activity patterns and behavioral variables like reaction times, learning biases, or reward processing (Koenig et al., 2008; Pedroni et al., 2011). Based on the latencies in which the TANCOVAs return significant, we could identify the cognitive processing step when the association manifest for H1, as described in Table 1.

To give a better understanding of our electrophysiological hypotheses, Spierer et al. (2013) have shown that inhibitory control training can lead to distinct changes in ERP components depending on the nature of the training task. For example, Go/NoGo training with consistent stimulus-response mappings enhances automatic inhibitory processes, reflected as early ERP modulations (ca. 80 ms) and increased parietal activation (Manuel et al., 2010). In contrast, a training that involves variable stimulus-response associations tends to reinforce more top-down inhibition processes, as indicated with training-induced modulation during later ERP components such as the N2, an effect interacting with the reward value of the stimuli (at ca. 200 ms post-stimulus; De Pretto et al., 2019; Najberg, Wachtl, et al., 2021).

For the behavioral hypotheses, a stronger emotional and attentional salience to appetitive cues should result in shorter reaction times for correct response trials, while the stronger inhibition conflict should result in a higher rate of commission errors (i.e., False Alarm rate). As for our electrophysiological hypotheses, H2a relates to baseline differences in the response to appetitive cues, while H2b relates to different changes in behavioral mechanisms induced by training.

## 2 Method

This study used data that were already collected but not yet accessed (bias control to protect against prior data observation: level 4). The data have been collected for another registered report receiving In-Principle Acceptance (ArticleID #563), which can be viewed at: https://osf.io/p763g?view_only=ed6417743e6e42e19a32ea43d0fc5baf. The Stage 1 of this manuscript can be found at: https://osf.io/2hwkn/.

All procedures were approved by the competent ethic committee, *Commission cantonale d’éthique de la recherche sur l’être humain* (Project-ID 2024-00118).

The eligibility questionnaires, stimuli, a demonstration of the gamified food GNG training, a showcase of the analyses performed via R, and the Supplementary Materials are available at our OSF study page: https://osf.io/mv6c5/.

### 2.1 Plan

For H1, given the nonlinearity of the relationship between the effect size of electrophysiological modulations and the associated behavioral modifications, a priori power calculations for our ERP hypotheses were not relevant. We, thus, relied on the behavioral hypotheses (H2) which required a larger sample size than those found in the ERP literature (Guttmann-Flury et al., 2019).

For H2, a priori power analysis computed with G*Power showed that 47 participants were required to reach a power of 90% for a one-sided correlation of 0.4 (weak to medium effect size; lowest effect size chosen for conservatism) between the sign-tracking bias and the outcome of interest (H2a: baseline performance; H2b: change in pre-post-training performance) with an alpha of 0.05. The alpha threshold was not corrected for multiple comparisons as both indexes of behavioral performance (i.e., reaction time and commission error rate) expressed different mechanisms of action and were interpreted independently.

The final sample size needed for our hypotheses was 47 participants.

### 2.2 General procedure

**Fig. 1.**
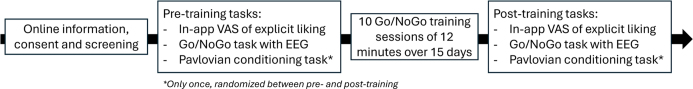
Study timeline.

Figure 1 summarizes the study timeline.

Participants were recruited via public advertisement. The inclusion criteria were 18- to 45-year-old healthy individuals who reported liking a majority of sugary drink types (i.e., at least 3 out of 5 on our custom Likert scales). Individuals with past or current diagnoses of eating disorders, current olfactory or gustatory impairments, dieting during the training phase, or with previous participation in food training studies were excluded.

After giving their consent and being screened for eligibility online, participants were invited to come to our laboratory. They downloaded our custom gamified training software—The Diner—either at home on their smartphone, or at the laboratory on a device lent to them in case of operating system incompatibility. The training software is first used to fill out visual analogue scales (VAS) of the target items’ explicit liking to both calibrate the food GNG task and training and to serve as baseline assessments.

At the laboratory, participants completed three computerized behavioral tasks: either a Sequential Learning or a Pavlovian conditioning task (randomized order), a Stimulus-Response Compatibility task, and a GNG task during a 64-electrodes EEG recording. The GNG task used the same items as the food GNG training. Only the Pavlovian conditioning and GNG tasks are relevant to this study.

Participants then trained at home to the food GNG for 10 sessions over 15 days, 12 minutes per session. At the end of the 10 sessions, the VAS of explicit liking of the trained items were filled again as post-training measurements.

Participants then came back to the laboratory for a post-training session, including the same tasks as the first session, but with either the Sequential Learning or Pavlovian conditioning task based on the randomized order.

Participants received 135 CHF with additional monetary gain based on their performance at the tasks as compensation. Participants who did not complete the training (i.e., no post-training VAS of explicit liking) were excluded from the study and compensated pro rata temporis.

### 2.3 Stimuli

#### 2.3.1 Sugary drink pictures

Fifty-seven pictures of sugary drinks representing the Swiss market were professionally taken on a white background and defined into nine types. Overall, this includes: 8 artificial energy drinks, 5 natural energy drinks, 4 iced coffee, 6 milky drinks, 16 citrus sodas, 3 colas, 2 other sodas, 2 kombucha, and 11 iced teas.

Sugary drinks were chosen as our target appetitive food items as they are easily recognizable to our target population and thus allow for a sensible baseline of self-reported explicit liking.

#### 2.3.2 Calibration algorithm

To categorize sugary drinks as either “Go” or “NoGo,” a calibration algorithm was designed to ensure that both Go and NoGo categories had the same number of items and similar baseline liking while keeping the same types of sugary drinks inside a given category (e.g., Coca-Cola vs. Pepsi) to avoid potential generalization effect. This categorization algorithm was run after the first VAS of explicit liking inside the training software. Its details are described in the Supplementary Materials on our OSF study page.

### 2.4 Pre-post-training tasks

#### 2.4.1 VAS of explicit liking

Within the training software, before and after the training, participants rated on VAS in random order all 57 sugary drink items from 0 (“not at all”) to 100 (“very much”) according to the question ‘Imagine drinking this, how much do you like it?’. A hashed blue line was displayed to point to the middle of the scale. These scales are identical to previous papers in our group to capitalize on their findings (Najberg, Rigamonti, et al., 2021; Najberg et al., 2023).

#### 2.4.2 Pavlovian conditioning task

**Fig. 2.**
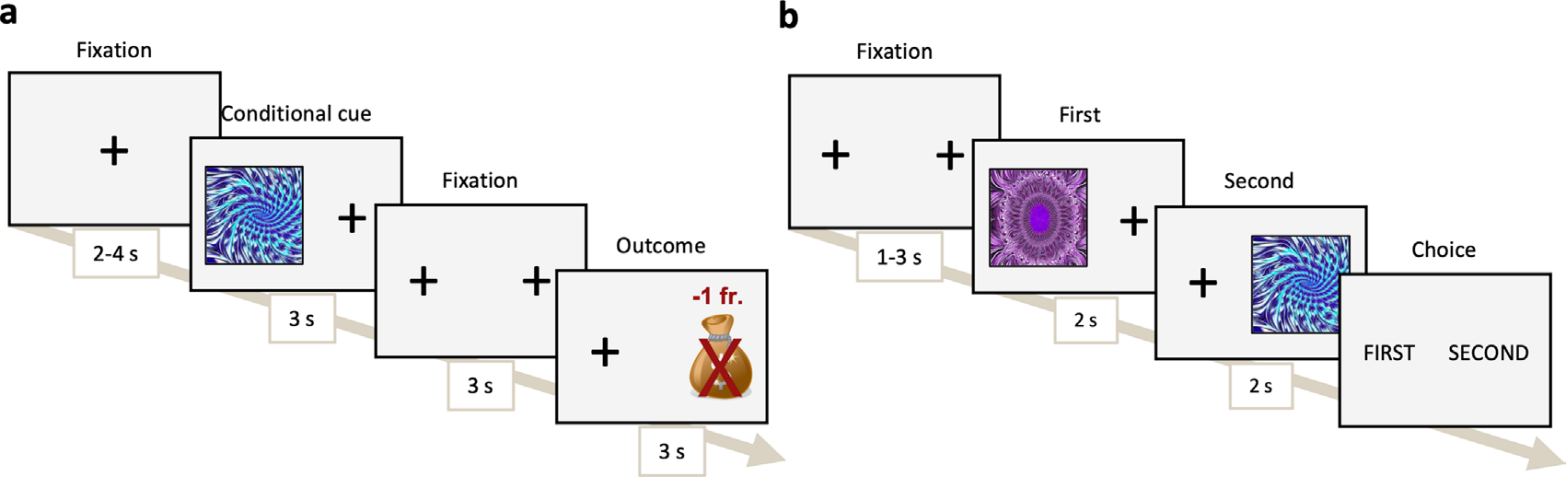
Schema of the Pavlovian conditioning task, describing the (a) Pavlovian conditioning and (b) Forced choice task timeline.

A timeline of the tasks can be found in Figure 2.

Participants completed a Pavlovian conditioning task (Schad et al., 2020) to assess their sign-tracking bias. The complete task consisted of the Pavlovian conditioning task and the Forced choice task.

##### 2.4.2.1 Pavlovian conditioning task

The Pavlovian conditioning task consisted of 80 trials, in which five fractal images (Conditioned Stimulus; CS) were associated with five different reward values (Unconditioned Stimulus; US): two negative values of -2 CHF and -1 CHF, one neutral value of 0 CHF, and two positive values of +1 CHF and +2 CHF. The identity of the fractal (CS) predicted the reward value (US). At each trial, a fractal stimulus was presented on the right or left side (randomized across trials) of the screen for 3 seconds, followed by 2 fixation crosses at the two potential stimulus locations during 3 seconds. The reward value was then presented on the opposite side. Participants were instructed to observe the stimulus and memorize the fractal-value pairs.

We recorded the participant’s fixation time on the various locations (CS, US, and background) through the SMI RED 500 eye tracking system, recording binocularly at 500 Hz.

To measure the sign-tracking bias of each participant, we followed the work of Schad et al. (2020). We first calculated a gaze index for each participant, defined as the difference between the proportion of fixation time directed at the conditioned stimulus (CS) and the proportion directed at the unconditioned stimulus (US) location during the third second of CS presentation (gaze index = p(CS) − p(US)). A gaze index of 1 reflects exclusive fixation on the CS, whereas a value of -1 indicates exclusive fixation on the US. Subsequently, for each participant, a linear regression was performed to predict the gaze index based on the reward value of the CS (that is, −2 CHF, −1 CHF, 0 CHF, +1 CHF or +2 CHF) following this formula:

p(CS)−p(US)=β1×CS reward value+ɛ

β1 is defined as the sign-tracking bias, with higher values indicating stronger sign-tracking tendencies.

##### 2.4.2.2 Forced choice task

To check whether the association between the US and CS took place during the previous phase, participants then performed a forced choice task of 30 trials in which they had to choose the CS with the highest associated value between two sequentially presented CSs. They were instructed to choose the fractal with the highest value and that they would receive 10% of the reward value associated with the chosen CS. Stimuli were presented one at a time for 2 seconds, after which the participants selected the first or second stimulus. Each possible CS pairing was presented 3 times in a randomized order. Participants were asked to respond faster if their response was more than 2 seconds.

#### 2.4.3 GNG task

**Fig. 3.**
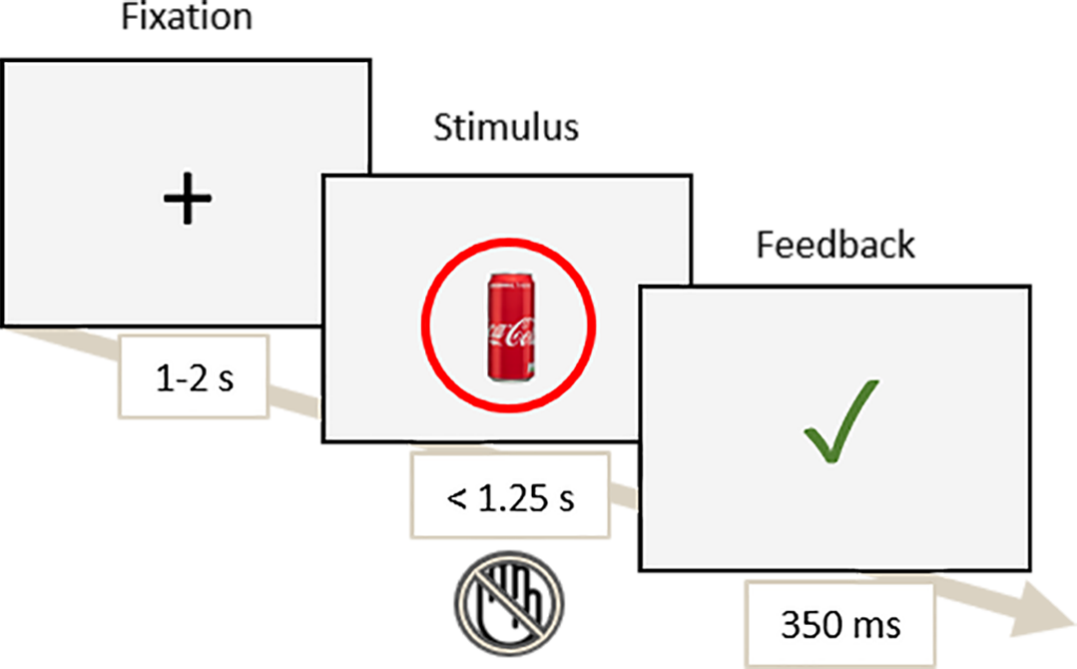
Timeline of the Go/NoGo task.

A timeline of the task can be found in Figure 3.

Participants were instructed to press as fast as possible on a response box to the items circled in green (“Go” trials, 60% of the trials) and withhold their response to the items circled in red (“NoGo” trials, 40% of the trials). The red and green cues were presented for 300 ms, and 100 ms post-stimuli’s onset. A total of four blocks of 200 trials each (120 Go and 80 NoGo per block; 800 trials total) were completed by the participants.

If a participant responded to a target item but was above the current auto-adaptive Reaction Time Threshold (RTT; i.e., 1.10*median of the current block’s RTs), then the feedback “Too late” was displayed. If a participant correctly withheld their response to a “NoGo” item (i.e., Correct Rejection) or responded to a “Go” item before the RTT (i.e., Hit), a green checkmark was displayed. If a participant erroneously responded to a “NoGo” item (i.e., False Alarm) or did not respond to a “Go” item (i.e., Miss), an “X” feedback was displayed. This procedure allows for maintaining a stable level of difficulty across participants and blocks.

### 2.5 Food GNG training

The GNG training task and its gamification followed the same procedure as Najberg et al. (2023), but without the neutral items, to capitalize on an already validated procedure. A demonstration of the training software can be found on our OSF page.

To ensure response potency (i.e., a high pre-activation of motoric response) during the training, 70% of the trials consisted of Go items, and 30% of NoGo items. This Go/NoGo proportion also strengthens the effect of the intervention, as the devaluation effect seems to appear when the NoGo rate is rare (Chen et al., 2016).

The reaction time threshold (RTT) of the Go items in this task is progressive. After 6 successful trials, the RTT increases of a level (Table 3). The RTT is not challenging at first to result in plenty of successful trials, a parameter strengthening the devaluation effect, and then increases until the participant repeatedly fails the trials to both load the inhibitory control and maximize the engagement to the gamified training.

**Table 3.** Difficulty level of the gamified Go/NoGo training task.

Difficulty level	1	2	3	4	5	6	7	8	9	10	11	12	13	14	15	16	17	18
RTT in seconds	1.1	1	.9	.8	.725	.675	.625	.575	.55	.525	.5	.475	.4525	.43	.407	.387	.36	.33

### 2.6 EEG processing

#### 2.6.1 Data collection

A 64-channel electroencephalogram was recorded at a sampling rate of 1,024 Hz with a Biosemi ActiveTwo system referenced to the common mode sense-driven right leg (CMS-DRL) ground placed on each side of the POz electrode. This circuitry consists of a feedback loop driving the average potential across the montage as close as possible to the amplifier zero (cf. the Biosemi website for a diagram). The Cartool software (Brunet et al., 2011) was used for visualization and importation/exportation of the raw and processed EEG data.

#### 2.6.2 Event-related Potentials (ERP) pre-processing

The pre-processing followed the EEGlab-based (Delorme & Makeig, 2004) autoERP toolbox (Najberg et al., 2020). This pipeline: 1) referenced the raw data to Cz, 2) apply band-pass filtering between 0.5 and 40 Hz, 3) removed sinusoidal noise at 50 and 100 Hz (Cleanline, https://www.nitrc.org/projects/cleanline), 4) filtered non-sinusoidal noise using Artifact Subspace Reconstruction (Chang et al., 2018; Mullen et al., 2015), 5) removed blinks detected during the stimulus onset (BLINKER plugin; Kleifges et al., 2017), 6) epoched the data to 100 ms before onset to 700 ms after onset, 7) applied baseline (i.e., all datapoints before onset) correction on the whole epoch range to correct for any remaining signal drifts, 8) interpolated identified bad channels using multiquadric interpolation (EEGInterp; Jäger & Buhmann, 2018), 9) excluded epochs with jumps of more than 30 µV from one-time frame (TF) to the next in at least one electrode, 10) excluded epochs with at least one TF with a voltage larger than 80 µV in an electrode, 11) averaged the epochs for each participant to create the ERPs, and 12) re-referenced the ERPs to the common average reference.

The targeted epochs were successful “NoGo” trials (i.e., correct rejection trials) at the GNG task.

### 2.7 Analysis plan

All tests were computed using R base functions with default arguments if not specified otherwise. Cohen’s d was computed using the DescTools package (Signorell, 2023). For behavioral analyses, Bayes Factors (BF_01_) between the model with and without the term of interest were computed using the BayesFactor R package (Morey & Rouder, 2018) with default priors and reported alongside *p*-values (independently if the result is significant or not). Please refer to the package manual for details on the priors (https://cran.r-project.org/web/packages/BayesFactor/BayesFactor.pdf).

For the following sub-sections, the data exclusion procedure followed the same order as the rules listed below. So first the global data exclusions, then the positive controls eventually leading to exclusions, then the exclusions specific to each hypothesis, eventually leading to different sample sizes at each hypothesis.

The behavioral analyses’ R script can be found on the OSF study page.

#### 2.7.1 Global data exclusions

As this study relied on the food cues to be appetitive, only items rated 60 or above to the pre-training VAS of explicit liking were considered for all analyses and positive controls.

We rejected participants not participating in the GNG task or not understanding its rules, as indexed by a rate of False Alarm above 70% or a rate of Miss above 20% at either the pre- or post-training (threshold based on our previous publications: Hartmann et al., 2016; Najberg, Rigamonti, et al., 2021).

We also rejected participants who did not learn the cue–reward pairings at the Pavlovian conditioning task, as defined by an error rate above 10% in the Forced Choice task.

We defined as outliers and excluded participants outside a 3*MAD range around the median of the sign-tracking bias, as this index is used for all subsequent positive controls and hypotheses.

#### 2.7.2 Positive controls

No correlation between the sign-tracking bias and the participants’ average pre-training VAS of explicit liking should be observed. Otherwise, the potential effect of the sign-tracking bias would not be distinguishable from the baseline explicit liking. If a correlation above 0.4 were to be observed, participants increasing the correlation would have been excluded until reaching this threshold.

To ensure sufficient variability in the sign-tracking bias, its standard deviation (sd) must be at least 15% of the observed range (max–min) in the sample. This conservative threshold provides a standardized measure of dispersion. If this condition were not met, we would have acknowledged that the variability in the metric is too limited to meaningfully distinguish between sign- and goal-trackers.

For H1b and H2b who are investigating pre-post-training modulations, we needed to ensure that the training did induce an effect. We verified that with a Cohen’s d of at least 0.4 to the pre- versus post-training average VAS of explicit liking of the trained NoGo items. In case of a lower Cohen’s dz, participants lowering the effect size would have been excluded until reaching this threshold. These exclusions would only have been considered for the corresponding hypotheses.

In case the exclusions performed to respect one positive control would have unbalanced the other, random exclusions of increasing set of participants would have been computed until finding the combination that allows to respect both positive controls with the minimal number of exclusions. These exclusions would not have been the object of re-recruitment in case we fell below the planned sample size to avoid circularity (i.e., having the exclusion thresholds being constantly updated after re-recruitment, eventually transforming previously excluded participants into included ones).

#### 2.7.3 H1a) Sign-tracking bias influences the baseline neural responses

We excluded participants without ERP in their post-processed signal, as determined by the visual inspection of a lab engineer with 14 years of experience in EEG recording and ERP processing blind to this study’s hypotheses.

Topographic Analyses of Covariance (TANCOVAs) were performed for each time frame (TF) on pre-training event-related potentials (ERPs) using the RAGU software (Koenig et al., 2011) (see Supplementary Materials for more details). The analysis used the sign-tracking bias as the covariate of interest. TANCOVAs were computed with 5,000 randomizations under the null hypothesis that no relationship exists between scalp field potentials and the covariate (i.e., the sign-tracking bias). The permutation was conducted across participants, by randomly shuffling the covariate values across subjects while keeping their ERP data fixed. Statistical significance was determined by comparing the observed Global Field Power (GFP) to the empirical null distribution, and we applied a cluster-based criterion (12 consecutive time frames with *p* < 0.05) to control for multiple comparisons across time. This minimal duration threshold was determined as the shortest duration of consecutive significant time-points that can be expected under the null-hypothesis (shuffled data) with a probability of 0.05 (Koenig & Melie-García, 2010; Koenig et al., 2011; Nichols & Holmes, 2002).

The GFP of the covariance maps at each time point served as the test statistic, quantifying the spatial strength of the EEG–covariate relationship. GFP is calculated as the square root of the mean of the squared potentials across all electrodes, centered around their average potential for each time point. Mathematically, it is expressed as:

$$\text{GFP}\;\;=\sqrt{\frac{1}{N_{e}}\sum_{i\;=\;1}^{N_{e}}{\left(v_{i}-\bar{v}\right)}^{2}}$$

where *N_e_* is the total number of electrodes, v represents the potential at electrode, and *v_i_* is the average potential across all electrodes. This calculation provides a reference-independent measure of the magnitude of scalp field variations, ensuring robust interpretation. The GFP values were also used to create the null distribution through randomization procedures. Covariance maps identified as significant were then submitted to source localization analyses to estimate the underlying neural generators (see Supplementary Materials for more details).

For each participant at each condition, the number of interpolated electrodes, percentage of rejected epochs, and number of kept epochs were reported as a measure of data quality.

#### 2.7.4 H1b) Sign-tracking bias influences the changes in neural responses after training

The same procedure as H1a was performed, except that only trials of items categorized as NoGo during training were considered, and that the TANCOVAs were computed on the within-subject factor Session (pre- vs. post-training) on top of having the sign-tracking bias as the covariate.

#### 2.7.5 H2a) Sign-tracking bias influences the baseline behavior during a GNG task

This hypothesis includes two indexes of performance computed and interpreted separately: the commission error rate (i.e., the number of erroneous responses divided by the number of total NoGo trials) and the average response time of correct responses at the GNG task. Participants outside a 3*MAD range around the median of either index were considered distribution outliers and removed from the respective analysis.

We expected a significant positive Pearson correlation between the sign-tracking bias and the commission error rate, meaning that STs would have more difficulty inhibiting their response in front of appetitive cues than GTs. We also expected a significant Pearson’s correlation between the sign-tracking bias and the average response time of correct responses, meaning that STs would present heightened emotional and attentional salience in front of appetitive cues than GTs.

The split-half reliability of the participants’ average response times and commission error rate were reported alongside this hypothesis’ results.

#### 2.7.6 H2b) Sign-tracking bias influences the changes in behavior after GNG training

The same procedure as H2a was performed, except that only trials of items categorized as NoGo during training were considered, and the indexes of performance were transformed into pre-post-training deltas.

The split-half reliability of the participants’ average response times and commission error rate were reported alongside this hypothesis’ results for both pre- and post-training measures.

## 3 Results

### 3.1 Population and data quality

**Fig. 4.**
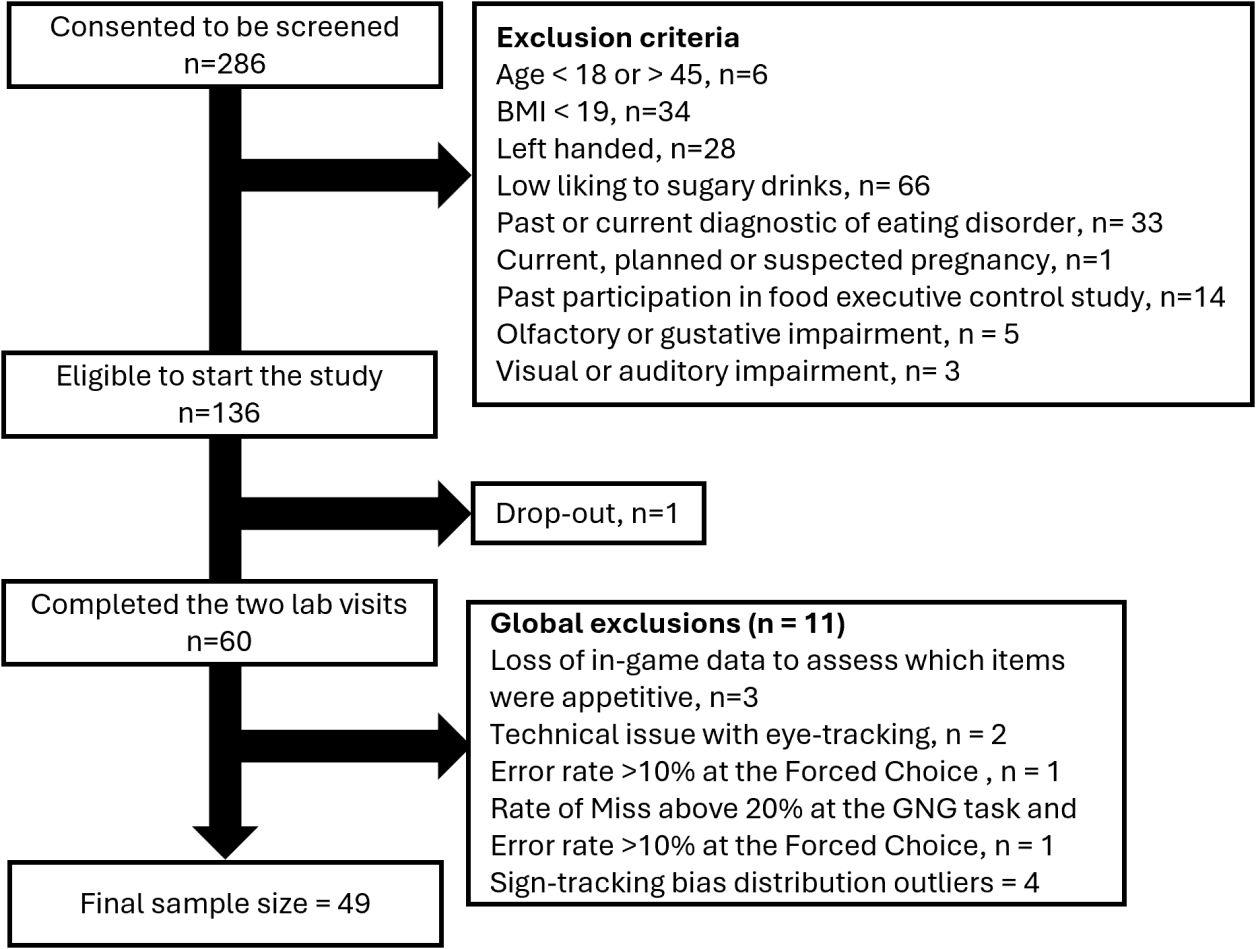
Sample size progression.

A total of 286 participants consented to the form online, 136 were eligible after the online screening, 61 participants came to the laboratory to participate in the first session, and one participant dropped out before the second visit, resulting in a total of 60 participants who completed the study (age = 25.2 years old ± 4.3; F:M Gender Ratio = 0.67, baseline (pre-training) explicit liking = 69.1 ± 14.0). After excluding participants due to technical loss of essential data and global exclusions’ rules, 49 participants were included in all hypothesis testing (target sample size: 47; see Fig. 4 for the sample size progression).

In this sample, all positive controls were respected, resulting in no further exclusions. The correlation between the baseline (pre-training) explicit liking and sign-tracking bias was small (*r* = 0.18), the variance of the sign-tracking bias was sufficient for its interpretation (18.3% of the range), and the Cohen’s d of the pre- versus post-training explicit liking was large (*d* = 0.91). Details can be found in the Supplementary Materials.

Despite the reduced number of epochs resulting from the exclusion of items with an explicit liking score below 60, no EEG data were excluded from a lack of ERP by our blind reader (see Table 4).

**Table 4.** EEG pre-processing results.

n = 49	Mean ± SD
Number of interpolated electrodes	9.60 ± 5.71 (15%)
Percentage of epochs with 80 µV artifacts	4.11 ± 19.92
Percentage of epochs with 30 uV jumps	0 ± 0
Number of kept epochs	153.16 ± 78.91

### 3.2 H1) Association between the sign-tracking bias and ERP

**Fig. 5.**
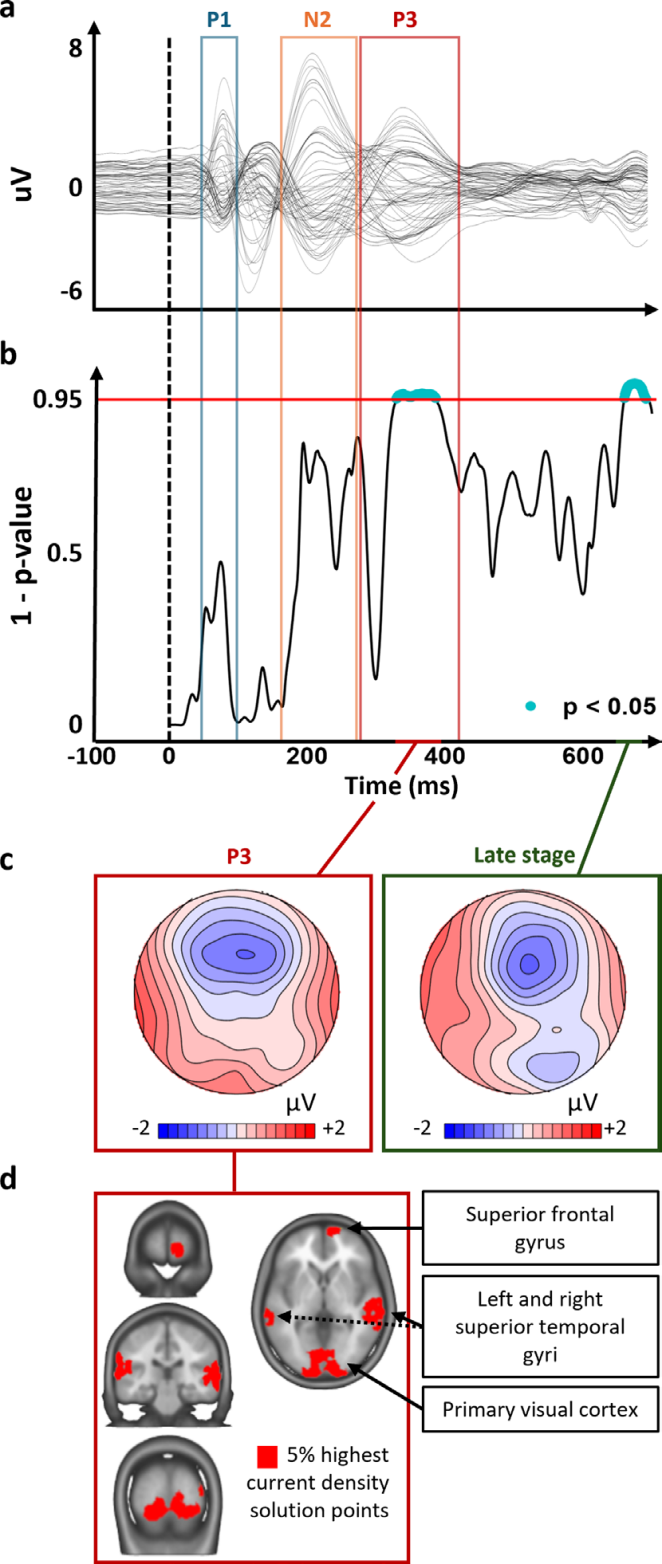
Covariance of ERP topography during successful inhibition of appetitive stimuli at baseline with sign-tracking bias (H1a). (a) Grand-average ERP. The amplitude (y-axis), the time (x-axis), and all components interval (P1, N2, and P3: blue, orange, and red square respectively) are represented. (b) Moment-by-moment significance levels of the TANCOVA are represented at each ms (x-axis). The height of the area indicates the significance level (Inverted *p*-values; y-axis; blue when significant) of the TANCOVA. The alpha threshold (0.05) is indicated with a red horizontal line. (c) Covariance map. Positive (red) and negative (blue) amplitudes are represented at the scalp-level. (d) The source points with the 5% highest current density at the source-estimation analyses during the P3 mapped onto the average MRI brain. The “late stage” component is not represented to avoid over-interpretating non-hypothesized components.

**Fig. 6.**
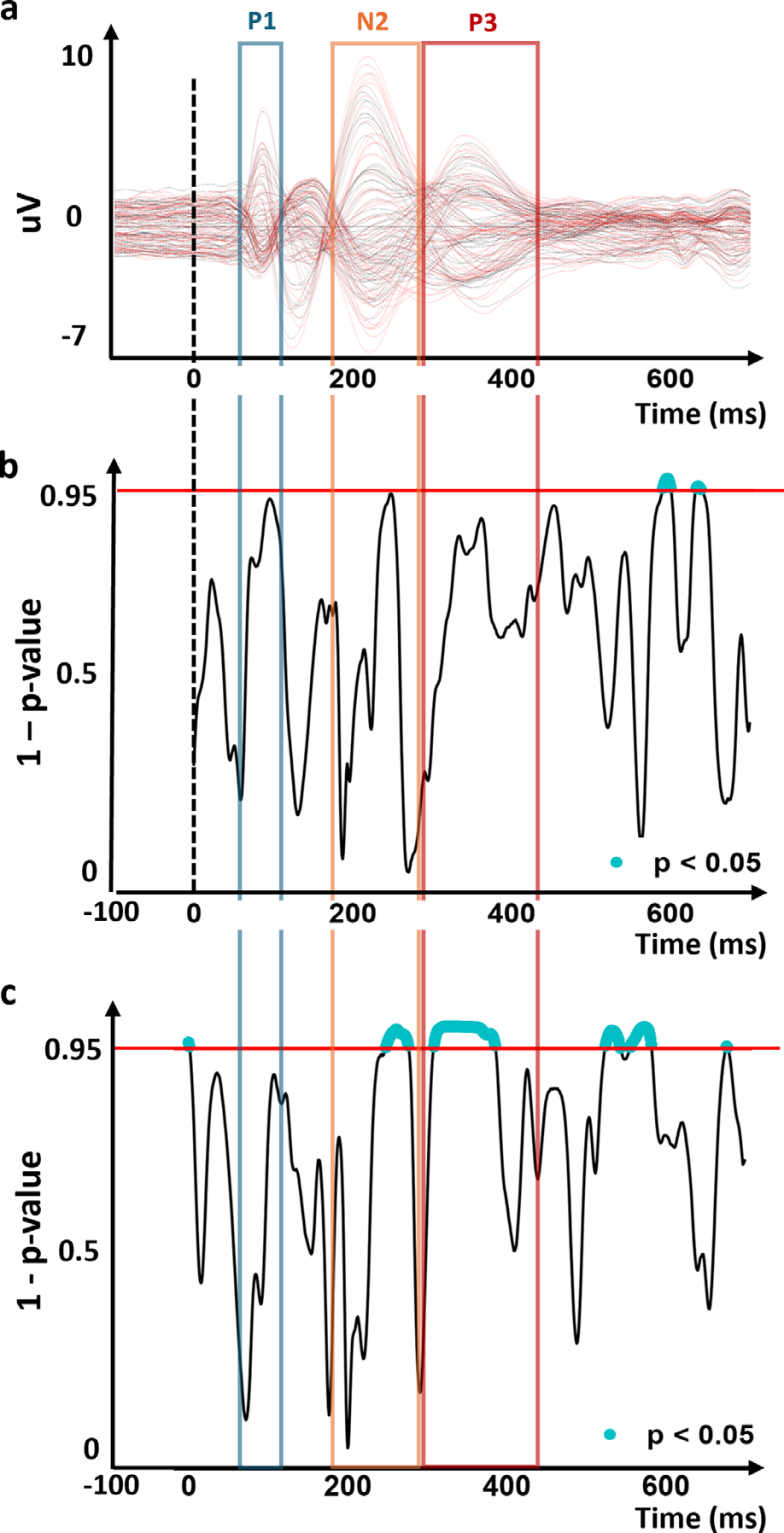
Covariance ERP topography during successful inhibition of appetitive stimuli for the pre- versus post-training with sign-tracking bias (H1b). (a) Superimposed pre-training (black) and post-training (red) grand-average ERPs. The amplitude (y-axis), the time (x-axis), and all components interval (P1, N2, and P3: blue, orange, and red square respectively) are represented. (b) Moment-by-moment significance levels of the TANCOVA are represented at each ms (x-axis). The height of the area indicates the significance level (Inverted *p*-values; y-axis; blue when significant) of the TANCOVA. The alpha threshold (0.05) is indicated with a red horizontal line. (c) Same as (b) but for the pre- versus post-training effect without the sign-tracking bias’s covariance.

Results of the ERP analyses are reported in Figures 5 and 6.

#### 3.2.1 H1a) Sign-tracking bias influences the pre-training (baseline) neural responses

The TANCOVA on the external variable sign-tracking bias at pre-training revealed significantly (*p* < 0.05) covarying EEG topographies in the time windows 329 and 385 ms after item onset (i.e., 58 consecutive significant time-frames; TFs), the period of the P3 ERP component. This was driven by larger positive amplitudes over the temporal electrodes and a more negative amplitude over fronto-central electrodes for high than low sign-tracking bias. Distributed electrical source estimations of the covariance ERP topographic map located its intracranial generators within the superior frontal gyrus, bilateral superior temporal gyri, and the primary visual cortex. This analysis reveals the relative contribution of intracranial brain sources covarying with the sign-tracking bias.

The TANCOVA further revealed significantly covarying EEG topographies in the time windows 659 and 690 ms after item onset (i.e., 32 consecutive significant TFs), during a late-latency ERP component. This was driven by more negative activity over central and parietal electrodes for higher sign-tracking bias. Because this component was not the focus of a registered hypothesis, no electrical source estimation or further discussion was focused on this period to avoid overinterpretation of post-hoc analyses.

#### 3.2.2 H1b) Sign-tracking bias influences the changes in neural responses after training

The TANCOVA on the external variable sign-tracking bias between pre- versus post-training revealed no significantly sustained covarying EEG topographies (i.e., above 12 consecutive TFs).

However, there were four instances of significant periods of interest when taking the pre- versus post-training effect only (i.e., effect of Session; no covariance with the sign-tracking bias): between 277 and 275 ms (late N2), 307 and 384 ms (P3), 523 and 541 ms, and lastly between 554 and 580 ms.

### 3.3 H2) Association between the sign-tracking bias and behavioral performance

#### 3.3.1 H2a) Sign-tracking bias influences the pre-training behavior during a GNG task

Two participants were flagged as distribution outliers and excluded from the commission error rate outcome, and none were flagged for the response time.

**Fig. 7.**
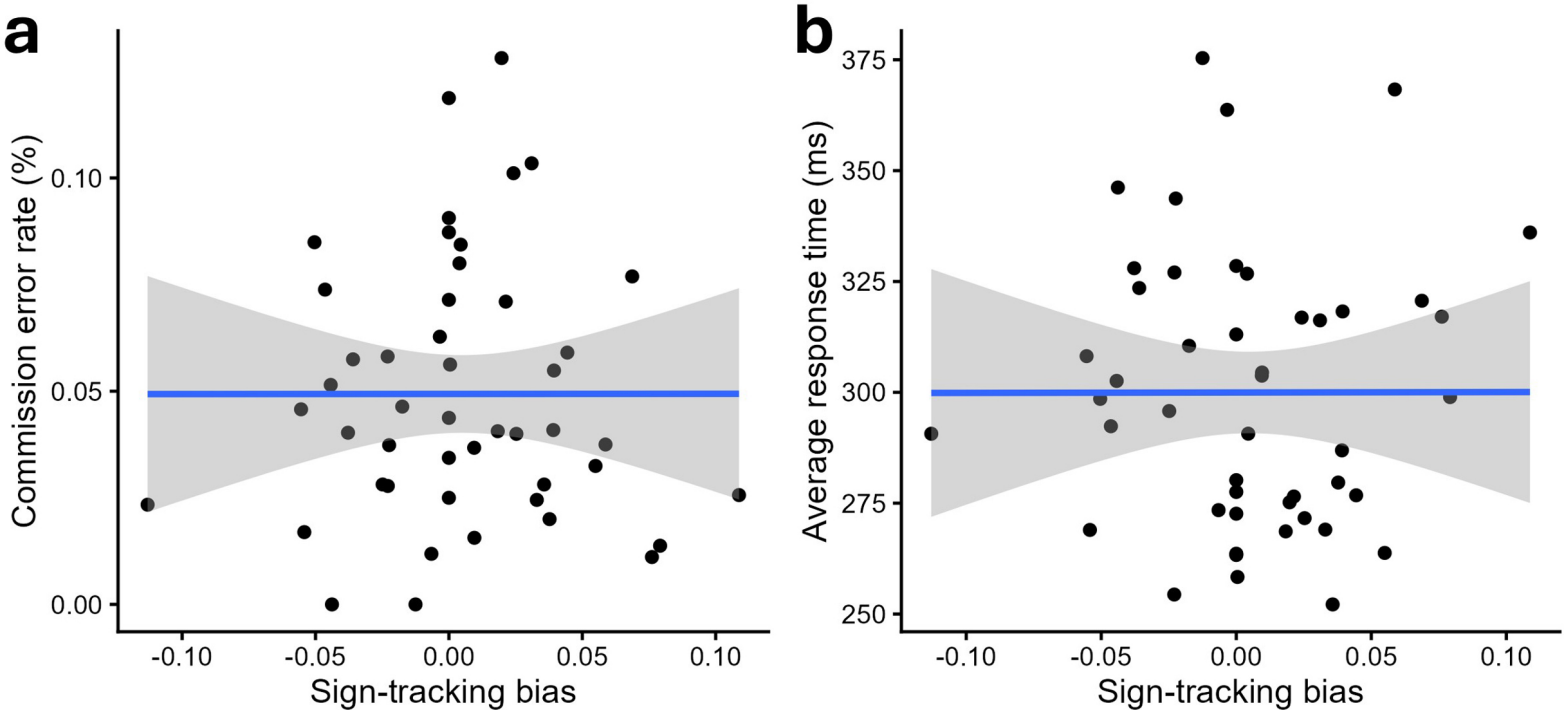
Scatter plot of the sign-tracking bias (x-axis) and the participants’ (a) commission error rate (%; y-axis) and (b) average response time (ms; y-axis). The linear regression is represented by the blue line, with its standard error indicated by the grey area.

Contrary to our hypothesis, there was no correlation between the error rate and the sign-tracking bias (*r* < 0.01, *t*[45] < 0.01, *p* = 0.998), nor between the response time and the sign-tracking bias (*r* = -0.02, *t*[47] = -0.11, *p* = 0.911) at pre-training (see Fig. 7). Bayes Factors against the null model indicate positive evidence for the null hypothesis (respectively: BF_01_ = 3.05 and BF_01_ = 3.10).

#### 3.3.2 H2b) Sign-tracking bias influences the changes in behavior with GNG training

Two participants were flagged as distribution outliers and excluded from the commission error rate outcome, and another two were flagged for the response time.

**Fig. 8.**
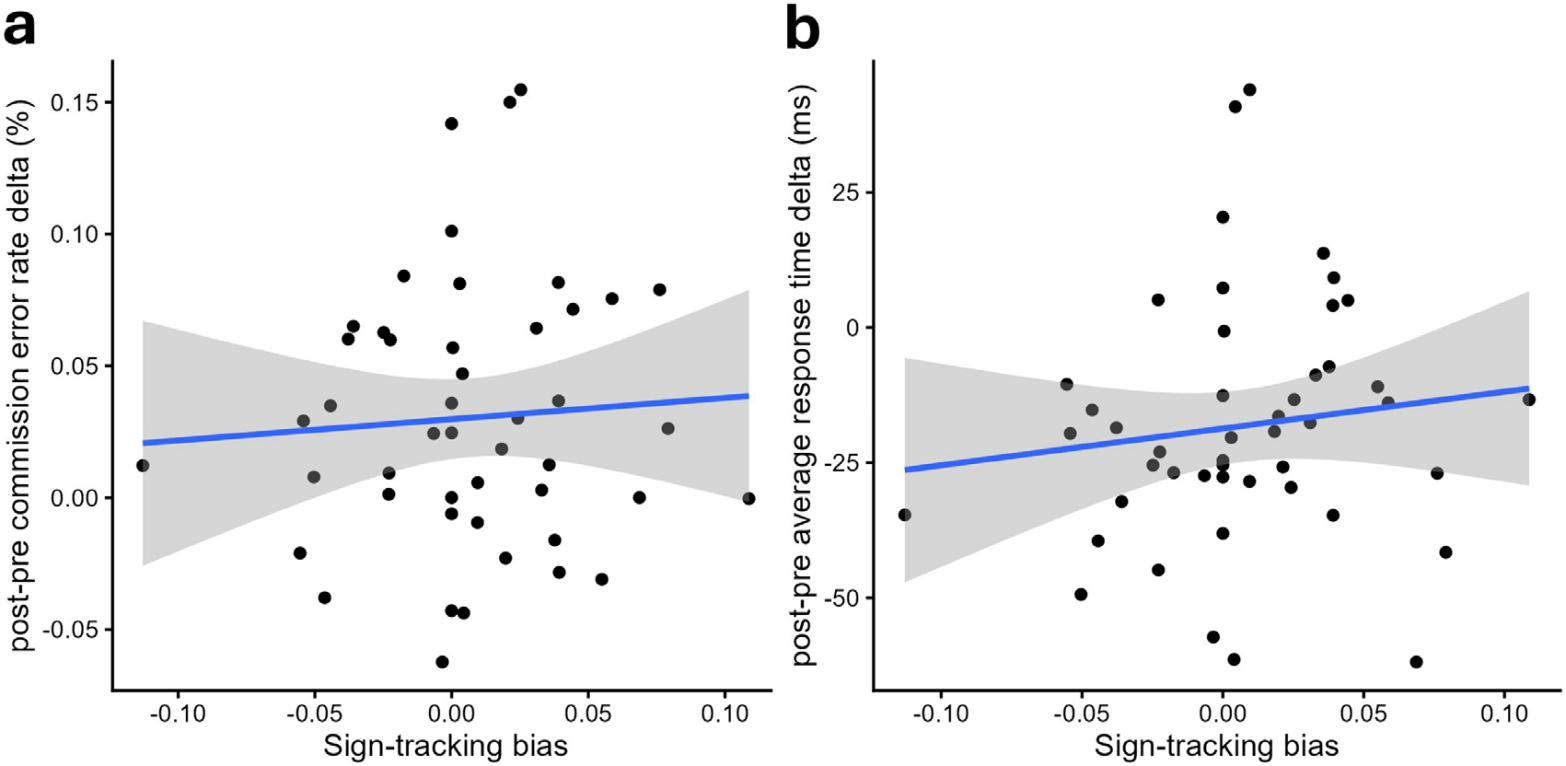
Scatter plot of the sign-tracking bias (x-axis) and the participants’ pre-post-training delta of their (a) commission error rate (%; y-axis) and (b) average response time (ms; y-axis). The linear regression is represented by the blue line, with its standard error indicated by the grey area.

Contrary to our hypothesis, there were no correlations between the pre-post-training delta of the error rate and the sign-tracking bias (*r* = 0.07, *t*[45] = 0.44, *p* = 0.660), nor between the pre-post-training delta response time and the sign-tracking bias (*r* = 0.08, *t*[45] = 0.57, *p* = 0.572; see Fig. 8). Bayes Factors against the null model indicate weak evidence for the null hypothesis (respectively: BF_01_ = 2.80 and BF_01_ = 2.64).

## 4 Discussion

In this electrophysiological interventional study, we capitalized on ERP topographic analyses of covariance to identify when and where individual differences in Pavlovian learning, as indexed by the sign-tracking bias during a Pavlovian conditioning task, covaried with brain functional responses to appetitive cues during a food Go/NoGo task (GNG) and with training-induced modulations. We further examined how sign-tracking bias covaried with the behavioral effects of the response training.

The registered hypotheses and positive controls ensured a robust interpretation of our results. Notably, the sign-tracking bias was assessed independently of baseline (pre-training) appetitive outcomes (*r* < 0.4), showed sufficient variability to allow meaningful interpretation (>15%), and the food GNG training was successfully administered to the participants, inducing a large pre- versus post-training devaluation (Cohen’s *d* > 0.4).

Overall, our findings confirmed some but not all of our hypotheses, as follows: i) H1a was confirmed: a sustained significant covariance between sign-tracking bias and pre-training ERPs to successful inhibition of appetitive cues during the P3 component; ii) H1b was not confirmed: no covariance of sign-tracking bias to ERP modulations induced by training, despite significant training-induced ERP changes during the N2 and P3 components; and iii) H2 was not confirmed: no relationship between sign-tracking bias and behavioral GNG performance (i.e., error rate and average response time) either at pre-training (H2a; *r* = 0.0, BF_01_ > 3) or pre- versus post-training (H2b; *r* = 0.1, BF_01_ < 3).

### 4.1 The sign-tracking bias is associated with pre-training ERPs

The TANCOVA at pre-training baseline revealed significant sustained covariance between the sign-tracking bias and the ERP during the P3 component. The P3 component is typically associated with the implementation of successful inhibition of response to NoGo cues (Antal et al., 2000; Nijs et al., 2009). This first shows that the sign-tracking bias is associated with the functional processes engaged during the food GNG task. To our knowledge, this is the first evidence that a value-related learning bias contributes to motor inhibition toward rewarding cues. Our finding further reveals that sign-trackers (STs; individuals tending to attribute motivational value to reward-predictive cues) did not differ from goal-trackers (GTs; individuals focusing on the relationship between cue and outcome) during the attentional salience (P1) or during the solving of the inhibitory conflict (N2) of appetitive cues, but during the inhibition of these cues (P3). As per our registered hypothesis, this indicates that the stronger the sign-tracking bias, the more individuals need to engage their inhibition, likely due to a stronger initial approach response elicited by the appetitive cues.

However, this electrophysiological result did not manifest at the behavioral level, which may be due to a ceiling effect. Indeed, young individuals typically have a good performance at the GNG task (Najberg, Wachtl, et al., 2021). The recruited population (25.2 years old on average) had a high average performance (5% ±3 error rate and 300 ms ±32 average response time), leaving little room for higher inhibition loading to translate into higher inhibition failures. Moreover, the overall commission error rate might not be sensitive enough to reveal differences in young healthy populations. Lastly, the average response time was not expected to change without modulations in attentional salience (i.e., no effect during P1).

The intracranial sources of the ERP covariance map had their maxima in the superior frontal gyri, a region involved in social and emotional cognitive processes (Bigler et al., 2007; Radua et al., 2009) and in the typical locus of interaction between the amygdala and prefrontal cortex (Adolphs, 2003). We also found an involvement of the bilateral superior frontal gyrus and the primary visual cortex, which is expected during high-level cognitive processes of visual cues. This frontal distribution supports that the sign-tracking bias covaries with the actual implementation of inhibition related to P3 and not general target detection which would show a maxima in parietal areas (Bokura et al., 2001; Hong et al., 2017). This corroborates that the electrophysiological signal during a food GNG task can detect the difference of emotional and cognitive processes induced by the sign-tracking bias.

### 4.2 The sign-tracking bias is not associated with training-induced ERP modulations

We found a main effect of training during the N2, P3, and later components, ensuring that our approach was sensitive to training-induced plasticity in the processes involved in the inhibition of response to appetitive cues. The food GNG training also induced a large behavioral devaluation effect (Cohen’s d = 0.91). Yet, the pre- versus post- training TANCOVA did not detect any covariation of the sign-tracking bias with training-induced ERP modulations.

These results suggest that if the effect of food GNG stems from updating the value of appetitive cues when paired with response inhibition (Veling et al., 2022), this mechanism is independent of the sign-tracking bias after training. Alternatively, our results might indicate that if sign-tracking bias interacts with GNG training’s mechanisms, the underlying processes may involve neural circuits not captured by the ERP components measured here, potentially reflecting distinct cognitive or motivational mechanisms beyond inhibitory control and reward processing (Duckworth et al., 2022).

Lastly, outcomes were assessed before and after a lengthy training, but no data were collected in-between: it is possible that the sign-tracking bias modulates the neurocognitive process taking place during the training without actually changing the post-training response.

### 4.3 Limitations

Our sample of healthy young adults may not be ideal for detecting individual differences in behavioral training outcomes, and their homogeneity and baseline performance may limit generalization to more diverse or clinical populations. In most studies assessing sign-tracking bias, monetary rewards are used irrespective of the underlying construct being investigated (Heck et al., 2025), while research employing a food-based task or exploring individual differences across reward modalities remains scarce (Lehner et al., 2017). In this context, it remains to be determined whether using a food reward instead of a monetary one might have provided a more sensitive measure of the specific mechanisms investigated in the present study. Lastly, continuous measures during the training period would allow capturing within-training dynamics and strategies, rather than reflecting only a post-training state.

## 5 Conclusion

This registered report revealed an association between the sign-tracking bias and functional activity during response inhibition implementation, but sign-tracking bias was not associated with changes in behavioral performance or training-induced electrophysiological modulations. Further studies should investigate the causal relationship between the sign-tracking bias and the neural response to the food GNG task to elucidate whether both theoretical frameworks overlap, additionally considering the domain-specificity of food and monetary reward.

## Supplementary Material

Supplementary Material

## Data Availability

Data and analyses scripts are available on our OSF page (https://osf.io/mv6c5/).
