# Supplementary material for: Sign-tracking bias is associated with the inhibition of motor response to appetitive food cues
Source: Imaging Neurosci (Camb). 2026 Feb 3;4:IMAG.a.1118. doi: 10.1162/IMAG.a.1118 (PMC12869319; doi:10.1162/IMAG.a.1118)
Supplement: Supplementary Material [file IMAG.a.1118_supp.pdf]

Supplementary Material for Stage 1 RR: "Electrophysiological correlates of Pavlovian learning bias and response training"

1. Stimuli generation of Pavlovian conditioning task

Fractals images have been generated using the AI tools DALL·E 2 and Stable Diffusion.

Prompts for fractals: "Collection of beautiful fractals in different colours."

After generating different sets of images using variations of these prompts, six crystals and five fractals with no obvious positive or negative connotation were selected.

The reward image was chosen from Pixabay with the keyword "money bag" (Pixabay, n.d., https://www.pixabay.com)

2. Pavlovian conditioning computations

To avoid a visual salience effect on the eye gaze at CS presentation, the gaze index is calculated using the last second of CS presentation.

The gaze index is computed as the proportion of fixation time on CS minus the proportion of fixation time on the US location.

Gaze index = p(CS) – p(US)

A linear regression is then performed for each participant on the gaze index with the true value of CS (-2 CHF, -1 CHF, 0 CHF, 1 CHF, 2 CHF) for each participant.

The computed regression coefficient is then used as a moderator of the intervention efficacy. A positive regression coefficient signifies a gaze attracted more to win-predictive than to loss-predictive CSs and thus corresponds to a sign-tracker tendency, whereas a negative coefficient signifies a gaze attracted towards the goal for expected wins more than for expected losses and expresses a goal-tracker tendency.

3. "Go" and "NoGo" items categorization algorithm

During the calibration phase, the following algorithm will ensure that both the "Go" and "NoGo" conditions will contain the same number of items and have an equivalent average liking within each participant.

Algorithm categorising the sugary drinks as "Go" or "NoGo"

1) While there are items still non-attributed to the "Go" or "NoGo" conditions, select the items of a random drink type category and attribute them to the condition with the least number of items.
2) Compute the average liking rate in both conditions. In the condition with the higher number of items, remove the items further away from the other condition's average. For example, if there are three more items in the "Go" than in the "NoGo" condition, and the "Go" condition is less liked than the "NoGo", then the three least liked "Go" items are removed.
3) Repeat the steps above 300 times and pick the result returning the smallest difference in liking between the "Go" and "NoGo" conditions, as assessed with a Cohen's d.

4. TANCOVA analysis details

The TANCOVA analysis (Koenig et al., 2008) used in our study is based on a multivariate linear model relating a continuous covariate (here, gaze behavior) to high-density ERP scalp field data at each time point (TF: time frame). Specifically, the TANCOVA approach does not involve computing a difference wave between two experimental conditions. Rather, it models the continuous covariation between a behavioral variable (e.g., gaze metrics) and the raw EEG scalp field at each time point. Each participant's full topography is entered into a general linear model, with the continuous covariate serving as the predictor. The key principle is that if brain sources covary with a continuous variable, this will be reflected as a linearly scaled addition to the scalp field. There is no subtraction of one condition from another; instead, each subject's EEG topography at each time point is regressed on the covariate. The resulting "covariance map" reflects the spatial pattern of neural activity that scales with the behavioral variable. While the term "ANCOVA" appears in the name for analogy with standard GLM approaches, the method is conceptually distinct from condition-based contrasts and instead captures parametric, graded relationships across participants.

Time-resolved analysis: The analysis is performed in a fully time-resolved manner, independently at each sample (e.g., each millisecond), with no temporal averaging or aggregation. This yields a time series of scalp maps whose topographic covariance with the covariate is tested at each time point.

ERP link and Table 1: The time windows presented in Table 1 in the main manuscript correspond to periods where the covariance between EEG topographies and the gaze covariate reached statistical significance, as determined by randomization testing of the GFP of the covariance maps. These intervals align with ERP components known to be functionally relevant.

Scalp field covariance: The TANCOVA output is a spatial covariance map representing the topographic configuration of EEG activity that systematically covaries with the covariate. This multivariate approach captures distributed neural effects and respects the spatial structure of EEG data, unlike univariate electrode-by-electrode testing.

5. Electrical Source Estimations

In case we were to analyse our ERP at the source-level for extra exploratory information, the following procedure will be applied:

Brain sources of ERP modulations will be estimated using a distributed linear inverse solution model (a minimum norm inverse solution) combined with the local autoregressive average (LAURA) regularization approach, which describes the spatial gradient across neighboring solution (Grave De Peralta Menendez et al., 2004; Menendez et al., 2001). LAURA enables investigating multiple simultaneously active sources and selects the configuration of active brain networks that better mimics biophysical behavior of neural fields. LAURA uses a realistic head model, and the solution space included 3005 nodes, selected from a grid equally distributed within the gray matter of the Montreal Neurological Institute's average brain. The head model and lead field matrix were generated with the spherical model with anatomical constraints (SMAC; Spinelli et al., 2000). As an output, LAURA provides current density measures; their scalar values were evaluated at each node. Assessments of the localization accuracy of this inverse solution by fundamental and clinical research indicate that the estimations and the results of their statistical analyses can be confidently interpreted at the resolution of the grid size (here 6 mm; e.g., Gonzalez Andino, Michel, et al., 2005; Gonzalez Andino, Murray, et al., 2005; Menendez et al., 2001; Michel et al., 2004).

5. Positive control results

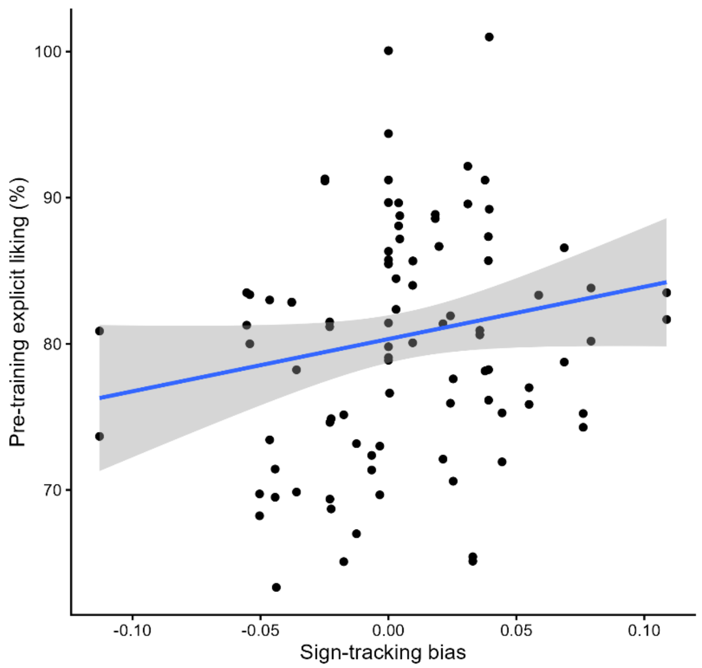

Supp. Figure 1 – *Scatter plot of the sign-tracking bias (x-axis) and pre-training explicit liking (%; y-axis). The linear regression (blue line) and its standard error (grey area) are represented.*

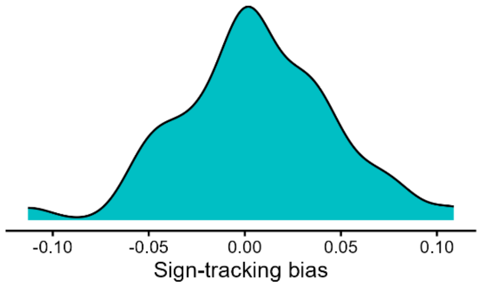

Supp. Figure 2 - *Distribution density of the sign-tracking bias*

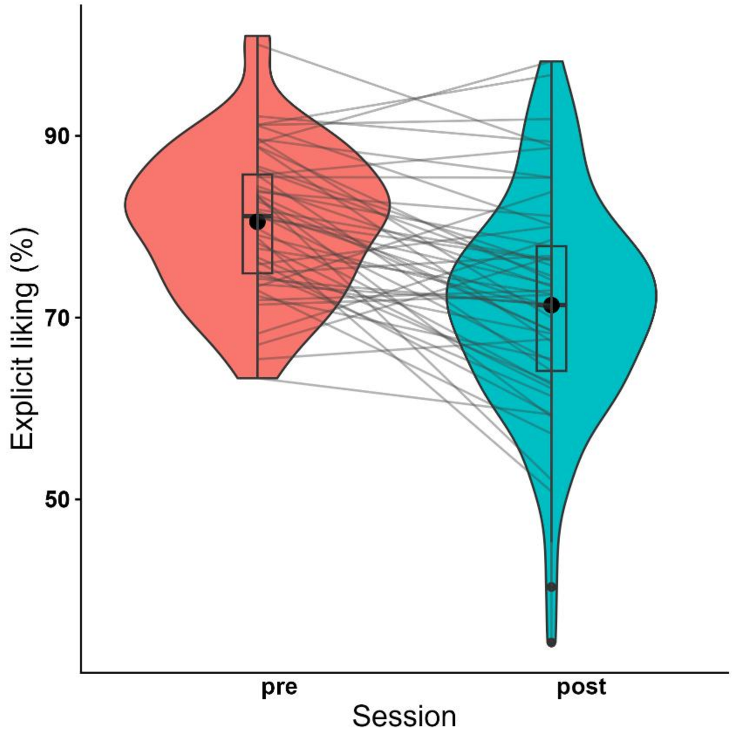

Supp. Figure 3 – *Violin plots representing the explicit liking of appetitive items (%; y-axis) at pre- and post-training (x-axis). Individual data points (grey lines), means (bold circle), distributions' density (violin), medians, first and third quartiles (horizontal bars), and the 1.5 inter-quartiles range (whiskers) are represented.*

References

Gonzalez Andino, S. L., Michel, C. M., Thut, G., Landis, T., & Grave de Peralta, R. (2005). Prediction of response speed by anticipatory high-frequency (gamma band) oscillations in the human brain. *Human Brain Mapping*, *24*(1), 50–58. https://doi.org/10.1002/hbm.20056

Gonzalez Andino, S. L., Murray, M. M., Foxe, J. J., & Menendez, R. G. D. P. (2005). How single-trial electrical neuroimaging contributes to multisensory research. *Experimental Brain Research*, *166*(3–4), 298–304. https://doi.org/10.1007/s00221-005-2371-1

Grave De Peralta Menendez, R., Murray, M. M., Michel, C. M., Martuzzi, R., & Gonzalez Andino, S. L. (2004). Electrical neuroimaging based on biophysical constraints. *NeuroImage*, *21*(2), 527–539. https://doi.org/10.1016/j.neuroimage.2003.09.051

Koenig, T., Melie-García, L., Stein, M., Strik, W., & Lehmann, C. (2008). Establishing correlations of scalp field maps with other experimental variables using covariance analysis and resampling methods. *Clinical Neurophysiology*, *119*(6), 1262–1270. https://doi.org/10.1016/j.clinph.2007.12.023

Menendez, R. G. D. P., Andino, S. G., Lantz, G., Michel, C. M., & Landis, T. (2001). Noninvasive localization of electromagnetic epileptic activity. I. Method descriptions and simulations. *Brain Topography*, *14*(2), 131–137. https://doi.org/10.1023/A:1012944913650

Michel, C. M., Murray, M. M., Lantz, G., Gonzalez, S., Spinelli, L., & Grave De Peralta, R. (2004). EEG source imaging. *Clinical Neurophysiology*, *115*(10), 2195–2222. https://doi.org/10.1016/j.clinph.2004.06.001

Spinelli, L., Andino, S. G., Lantz, G., Seeck, M., & Michel, C. M. (2000). Electromagnetic inverse solutions in anatomically constrained spherical head models. *Brain Topography*, *13*(2), 115–125. https://doi.org/10.1023/A:1026607118642
